# Norovirus Epidemiology in Community and Health Care Settings and Association with Patient Age, Denmark

**DOI:** 10.3201/eid2007.130781

**Published:** 2014-07

**Authors:** Kristina T. Franck, Jannik Fonager, Annette K. Ersbøll, Blenda Böttiger

**Affiliations:** Statens Serum Institut, Copenhagen, Denmark (K. T. Franck, J. Fonager, B. Böttiger);; University of Southern Denmark, Odense, Denmark (K. T. Franck);; University of Southern Denmark, Copenhagen (A. K. Ersbøll);; Lund University, Malmö, Sweden (B. Böttiger)

**Keywords:** norovirus, viruses, gastroenteritis, genotype, age distribution, hospitals, community-acquired infections, general practice, health care settings, molecular epidemiology

## Abstract

Norovirus (NoV) is a major cause of gastroenteritis. NoV genotype II.4 (GII.4) is the predominant genotype in health care settings but the reason for this finding is unknown. Stool samples containing isolates with a known NoV genotype from 2,109 patients in Denmark (patients consulting a general practitioner or outpatient clinic, inpatients, and patients from foodborne outbreaks) were used to determine genotype distribution in relation to age and setting. NoV GII.4 was more prevalent among inpatients than among patients in community settings or those who became infected during foodborne outbreaks. In community and health care settings, we found an association between infection with GII.4 and increasing age. Norovirus GII.4 predominated in patients ≥60 years of age and in health care settings. A larger proportion of children than adults were infected with NoV GII.3 or GII.P21. Susceptibility to NoV infection might depend on patient age and infecting NoV genotype. Cohort studies are warranted to test this hypothesis.

## Abstract 2

Norovirus GII.4 predominated in patients ≥60 years of age and in health care settings.

Norovirus (NoV) is a major cause of viral gastroenteritis (*1*) and a common cause of outbreaks of acute gastroenteritis in institutional settings, such as hospitals, nursing homes, and schools. Foodborne outbreaks of NoV infection are also common (*2**,**3*).

NoVs are positive-sense, single-stranded, non-enveloped RNA viruses (*4*). On the basis of amino acid or nucleotide sequencing of the polymerase and capsid regions, NoV can be divided into 6 genogroups (GI–GVI) and several genotypes. GI, GII, and GIV are human pathogens (*5**–**7*). Recombination events within a genogroup are common (*8*). Thus, genotyping of NoV should ideally be based on sequencing of the capsid and polymerase regions of the viral genome (*9*).

NoV sequences reported to the Foodborne Viruses in Europe Network come from mainly foodborne outbreaks or outbreaks in health care settings (*2*). Outbreaks in health care settings are most often caused by NoV genogroup II genotype 4 (GII.4) (*10**–**13*). The proportion of outbreaks caused by GII.4 is lower in non–health care settings (*2**,**3**,**12**,**14*). Elderly persons seem to be more susceptible to NoV infection (*15**,**16*). This susceptibility has been suggested to be genotype dependent (*3*).

The purpose of this study was to describe the distribution of NoV genotypes among infections in patients consulting a general practitioner (GP) or outpatient clinic, patients in health care settings, and patients in foodborne outbreaks. The association between NoV GII.4 and age of the patients in community and health care settings was also determined.

## Materials and Methods

### Patient Samples

The study included patients who had stool samples test positive for NoV during routine diagnostic virus analyses at the Department of Virology at Statens Serum Institut, Copenhagen, Denmark, during 2006–2010. This department serves as a reference laboratory and, throughout the study period, also served as the primary virus diagnostic laboratory for most GPs, outpatient clinics, and hospitals in Denmark. Information about sampling date, setting (i.e., hospital, GP, or outpatient clinic), age, and sex of the patients was obtained from the laboratory database. Samples from patients infected during suspected foodborne outbreaks of gastroenteritis were accompanied by special request forms at submission to the laboratory. Information regarding hospital admissions (dates and wards) during the study period was obtained from the Danish Health and Medicines Authority. Patients registered in the laboratory database as inpatients were excluded if hospitalization at the time of sampling could not be verified. Collection and registration of patient data were approved by the Danish Data Protection Agency (record nos. 2012–54–0046 and 2010–54–1076).

Using the personal identification numbers mandatory for all Danish citizens, we obtained postal addresses for patients ≥60 years of age who were positive for NoV and had a sample with an assigned genotype submitted from an outpatient clinic or GP. The addresses were used to determine if these patients were nursing home residents as of July 2013. Patients who had died before July 2013 were excluded because it was not possible to determine if they had been nursing home residents (n = 21).

Patient NoV samples were obtained from 3 settings. The first group consisted of inpatients and nursing home residents (referred to as health care settings), the second group consisted of patients consulting a GP or outpatient clinic (referred to as community settings), and the third group consisted of patients from foodborne outbreaks. The patients were from all 5 regions of Denmark.

Sampling and admission dates were used to estimate whether infections were nosocomial or community acquired. An infection was classified as community acquired if stool samples were obtained on the day of admission or the following day, nosocomial if samples were obtained on day 5 or afterwards, and indeterminate if samples were obtained between these 2 periods. Multiple samples were submitted from 1,060 patients. To avoid overrepresentation of patients chronically infected with NoV, only the first NoV-positive sample from each patient was included. During the study period, samples were continuously selected for genotyping. The intention was to type all samples from community settings, ≥1 sample from every hospital ward per month, and 1 sample from each foodborne outbreak, respectively, which yielded 2,231 samples.

### RNA Extraction

Stool samples were processed as 10% (wt/vol) suspensions in phosphate buffer solution, centrifuged at 4°C for 30 min at 3,400 *g*, and analyzed within 72 h of arrival. Nucleic acids were extracted by using MagNa Pure LC (Roche Diagnostics, Hvidovre, Denmark) and the Viral NA Small Volume Kit (Roche Diagnostics) according to the manufacturer’s instructions.

### Real-time Reverse Transcription PCR

NoV GI and GII were detected by real-time reverse transcription PCR (RT-PCR) by using the OneStep RT-PCR Kit (QIAGEN, Aarhus, Denmark) and primers and probes, as previously described (*17*). PCR conditions are shown in the Technical Appendix.

### NoV Genotyping

#### Polymerase RT-PCR

Polymerase gene sequences were obtained by using primers JV12Y-JV13 (*18*) or JV12BH-NVp110 (*18**,**19*) in 1 round of amplification. If PCR results were negative, a nested PCR was performed (*20*). Using the above-mentioned primers, we performed an RT-PCR with the OneStep RT-PCR Kit (QIAGEN) for the first-round PCR and AmpliTaq 360 DNA Polymerase (Applied Biosystems, Naerum, Denmark) for second-round PCR according to the manufacturers’ instructions. PCR conditions are shown in the online Technical Appendix.

#### Capsid RT-PCR

Capsid gene sequences were obtained by using a semi-nested GI-specific primer set (GIFF-1, GIFF-2, and GIFF-3 for a first-round PCR and GISKR [GIFFN and GISKR] for a second-round PCR), which amplified 305 bp of the GI capsid gene; or a semi-nested GII-specific primer set (G2FB-1, G2FB-2, and G2FB-3 for a first-round PCR and G2FBN [COG2F and G2SKR] for a second-round PCR), which amplified 299 bp of the GII capsid gene (*17**,**21**,**22*). Using these primers, we performed an RT-PCR by using the OneStep RT-PCR Kit (QIAGEN) for a first-round PCR and AmpliTaq 360 DNA Polymerase (Applied Biosystems) for a second-round PCR according to the manufacturers’ instructions. PCR conditions are shown in the online Technical Appendix.

### Sequencing

PCR products were prepared for sequencing by using Exo-SAP (GE Healthcare, Little Chalfont, UK) according to the manufacturer’s instructions. Both strands of DNA were sequenced by using an ABI 377 DNA Sequencer (Applied Biosystems) with the same primers used for RT-PCR and the Big Dye Terminator Kit 1.1 (Applied Biosystems).

### Sequence Analysis and Identification of Genotype

Sequence analysis and assembly were performed by using BioNumerics version 6.6 (Applied Maths, Sint-Martens-Latem, Belgium). Genotypes were assigned by using phylogenetic analyses (http://www.rivm.nl/mpf/norovirus/typingtool) (*6*). Genotyping was primarily based on the polymerase sequence. If this procedure was not successful, sequencing of the capsid genome was attempted. For some sample gene products, both regions were sequenced. If divergent genotypes were detected in the capsid and polymerase genes, the capsid genotype was used.

### Descriptive Analyses

Distribution of patients with respect to age and setting was initially determined by using all 3,848 samples. To obtain a representative picture of the distribution of circulating NoV genotypes and to avoid including several patients from the same outbreak, we included only the first sample from each clinic and ward within a calendar month (n = 1,612). The difference in age between patients with and without an assigned genotype was obtained for community and health care settings separately by using the Wilcoxon-Mann-Whitney test. The association between an assigned genotype (as the outcome) and age and sex (separately) was evaluated by using univariable logistic regression analysis. The association between genotype and age group was tested by using the Pearson χ^2^ test.

### Association between NoV GII.4 and Patient Age

The association between age and infection with NoV GII.4 was measured by using multilevel logistic regression analysis. Patients grouped within the same cluster (ward or clinic) are often more similar than randomly selected patients from different clusters. To account for this lack of independence between patients in clusters, a multilevel model was used that assumed a normal distribution of random effects. A total of 523 clusters (212 wards and 311 clinics) were included. The outcome was NoV genotype as the binary variable (GII.4 or non-GII.4). Three covariates were included in the analysis as fixed effects: age (<3, 3–19, 20–39, 40–59, and ≥60 years), setting (community or health care), and sex. Two interactions were considered of interest and were included in the analyses; these were the interactions between setting and age and between setting and sex. Backward elimination was used to exclude non-significant interactions by first removing the most non-significant interaction.

The mean cluster size was 3.73 (range 1–55). To evaluate the effect of a small cluster size, the analysis was repeated by including only clusters (i.e., wards and clinics) with ≥5 patients in the analysis. The analysis was also repeated by using logistic regression without any random effect on the descriptive dataset shown in Table 1 (i.e., first patient with an assigned genotype from each clinic and ward within a calendar month).

**Table 1:** Age and setting for 3,848 patients with stool samples positive for norovirus, Denmark, 2006–2010*

Age group, y	All patients, no. (%)	Descriptive analysis,† no. (%) patients positive for GII.4)‡

Community settings	Health care settings	Foodborne outbreaks	Community settings	Health care settings	Foodborne outbreaks
<3	680 (61)	36 (1)	NR	490 (51)	23 (57)	NR
3–19	113 (10)	15 (1)	NR	71 (27)	10 (50)	NR
20–39	145 (13)	76 (3)	NR	94 (62)	33 (64)	NR
40–59	93 (8)	240 (9)	NR	64 (64)	90 (89)	NR
≥60	86 (8)	2,196 (86)	NR	62 (82)	629 94)	NR
Total	1,117 (100)	2,563 (100)	168 (100)	781 (54)	785 (91)	46 (41)

*Only 1 sample per patient was included. GII, genogroup II; NR, not relevant. †Only 1 patient per clinic or ward per month was included. ‡Proportion of patients infected with novovirus GII.P4 or G.II.4 in each age group.

Stata software version 11.2 (StataCorpLP, College Station, TX, USA) and SAS version 9.3 (SAS Institute, Cary, NC, USA) were used for analyses. Significance was determined at p<0.05 and by using 2-sided tests.

## Results

During the 5-year study period, stool samples from 18,796 patients were submitted to the Department of Virology at Statens Serum Institut. A total of 4,056 patients were positive for NoV. After exclusion of patients with uncertain hospitalization status, 3,848 patients were included for further analysis (Table 1). These patients were from 230 wards in 60 hospitals in Denmark, 356 general practices or outpatient clinics, and 46 suspected foodborne outbreaks. A NoV genotype was identified for 2,109 patients. Of these patients, 1,713 had samples initially selected for genotyping. In 223 of the selected samples, a genotype was not obtained because of lack of sensitivity or sample material; genotyping was not attempted for 295 other samples.

A genotype based on sequence information from the polymerase and the capsid genes was obtained for NoVs in 349 (17%) samples. NoVs from 1,496 (71%) samples were genotyped by partial sequencing of the polymerase gene and NoVs from 264 (13%) samples were genotyped by partial sequencing of the capsid gene. Thus, a genotype was established for NoVs in samples from 204 (89%) wards, 59 (98%) hospitals, and 313 (88%) clinics. A genotype was established for NoVs in ≥1 sample from all foodborne outbreaks. The age distribution differed significantly between patients for whom an NoV genotype was identified and those for whom it was not (community settings: p = 0.002; health care settings: p<0.001. However, when we compared patients ≥60 years of age in community settings with patients <3 years of age, the proportion of genotyped NoVs in samples did not differ significantly (odds ratio 0.6, 95% CI 0.4–1.1, p = 0.1).

Among the 2,109 patients for whom the infectious agent had an assigned NoV genotype, 882 patients were from community settings, 1,070 were from health care settings, and 157 were from foodborne outbreaks. Patients from health care settings were further grouped into nosocomially infected patients (n = 539), patients with community-acquired infections (n = 248), patients with an indeterminate source of infection (n = 274), and nursing home residents (n = 9).

**Figure 1:**
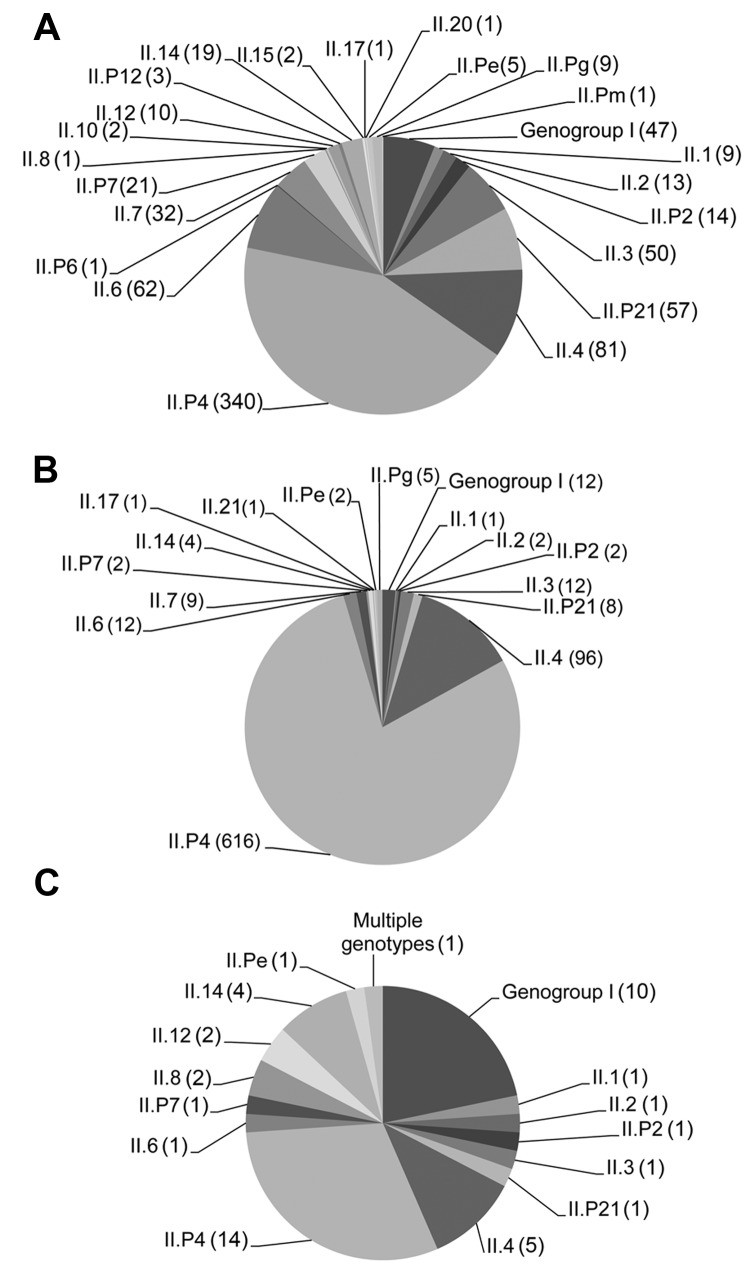
Distribution of norovirus genotypes of isolates from stool samples of A) patients in community settings (n = 781 samples), B) patients in health care settings (n = 785 samples), and C) patients in foodborne outbreaks (n = 46 samples), Denmark, 2006–2010. From each clinic and hospital ward, only the first sample with an assigned genotype per calendar month is included. Values in parentheses are numbers of isolates with a specific genotype or genogroup.

A total of 22 NoV capsid and 15 polymerase genotypes were detected among the genotyped samples. In patients from community settings, 20 capsid and 12 polymerase genotypes were detected, and 14 capsid and 8 polymerase genotypes were detected in NoVs from patients in health care settings. With the exception of GII.21, all NoV genotypes detected in health care settings were also detected in community settings. Among the samples from the 46 foodborne outbreaks, 15 NoV capsid and 11 polymerase genotypes were detected. The 2 most prevalent genotype combinations were GII.P21_GII.3 and GII.P7_GII.6, and the 6 most common genotypes were GII.P4, GII.4, GII.6, GII.3, GII.P21, and GII.7 (Figure 1). Clinics (n = 60) and wards (n = 63) that were represented with ≥5 patients with an assigned genotype had median proportions of NoV GII.4 of 57% (range 14%–100%) and 96% (range 17%–100%), respectively.

The age distribution differed considerably between patients from community and health care settings (Table 1). A significantly larger proportion of patients from health care settings were ≥60 years of age (2,196/2,563, 86%) (p<0.001). In contrast, patients from community settings were mainly children <3 years of age (680/1,117, 61%) (p<0.001).

### Descriptive Analyses

In these analyses, only 1 patient per calendar month from each GP, outpatient clinic, and ward was included (Table 1). Foodborne outbreaks were described on an outbreak level with only 1 sample representing each outbreak (n = 46 outbreaks).

**Figure 2:**
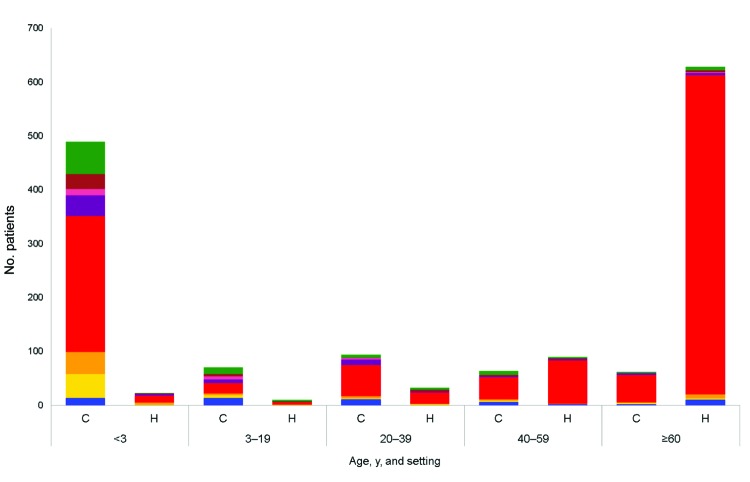
Distribution of norovirus genotypes by patient age and setting for 1,566 patients positive for norovirus, Denmark, 2006–2010. Only 1 patient from each general practice or outpatient clinic (n = 781 patients) and ward (n = 785 patients) within a calendar month were included (foodborne outbreak patients were not included). Inpatients and patients from nursing homes were grouped as patients from health care settings. Genogroup I (GI), blue; GII.P21, gold; GII.3, orange; GII.4 or GII.P4, red; GII.6, purple; GII.P7, pink; GII.7, brown; all other genogroup II, green. C, community; H, health care.

The distribution of NoV genotypes according to age and setting is shown in Figure 2. The distribution differed between community and health care settings. Although most patients from health care settings were infected with GII.4 (712/785, 91%), this genotype was detected in a significantly lower proportion of patients from community settings (421/781, 54%) (p<0.001). The proportion infected with GI was significantly higher in foodborne outbreaks (22%) than in community settings (6%) and health care settings (2%) (p<0.001). When samples positive for NoV GII.4 and GII.P4 were excluded, the proportion of GI was similar for those infected in community (13%) and health care settings (16%) but significantly higher for those infected in foodborne outbreaks (37%) (p = 0.001).

The proportion of children <3 years of age infected with NoV GII.3 or GII.P21 ranged from 11% to 25% during the study period. However, ≤3% of adults ≥60 years of age were positive for these genotypes. This difference was significant for each year studied (each year tested: p<0.001).

### Association between NoV GII.4 and Patient Age

**Figure 3:**
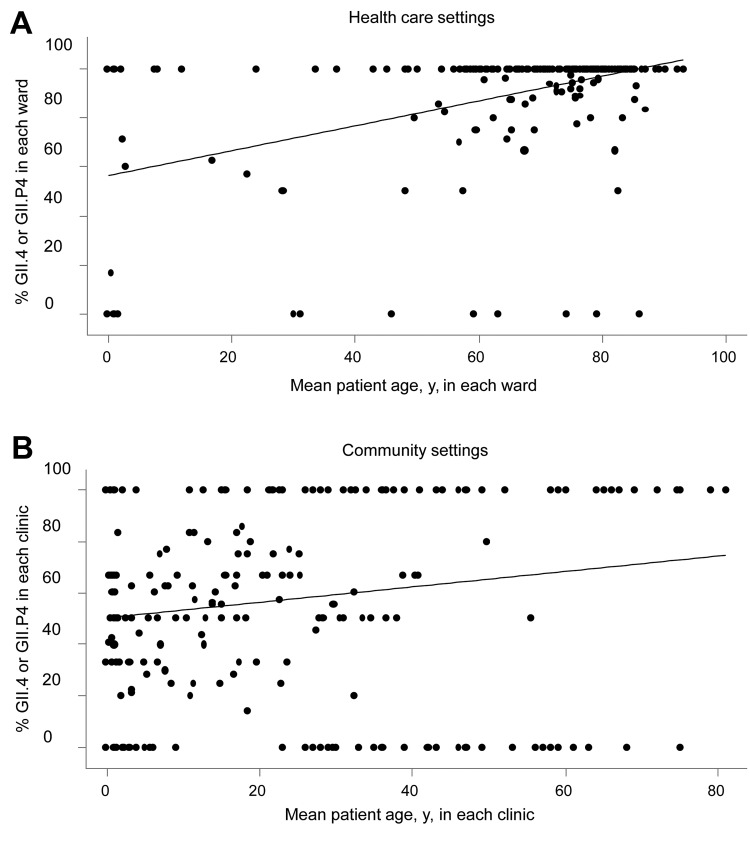
Proportions of norovirus genogroups GII.4 or GII.P4 with respect to mean age of patients with an assigned genotype in each hospital ward (A) (n = 212 wards, 1,070 patients) or clinic (B) (n = 311 clinics, 882 patients), Denmark, 2006–2010. Regression lines are indicated.

When we compared younger and older infected persons, we found a strong association between infection with NoV GII.4 and patient age ≥60 years in community and health care settings. This association was greater in health care settings than in community settings (Table 2) (p<0.001 for effect of age and setting). The mean proportion of NoV GII.4 within each ward or clinic with respect to the mean patient age is shown in Figure 3. The sensitivity analysis showed similar results regarding odds ratios (Table 2).

**Table 2:** Association between age and infection with norovirus GII.4, Denmark, 2006–2010*

Variable	All patients with an assigned genotype, n = 1,952					Sensitivity analysis 1, n = 1,299 patients			Sensitivity analysis 2, n = 1,566 patients	
	Total no. (%)†	No. (%) positive for GII.4‡	OR	95% CI		OR	95% CI		OR	95% CI
Community settings										
Age, y										
<3	558 (63)	291 (52)	0.60	0.39–0.91		0.46	0.25–0.86		0.63	0.40–1.00
3–19	80 (9)	21 (26)	0.19	0.10–0.37		0.09	0.03–0.24		0.22	0.11–0.43
20–39	114 (13)	73 (64)	Ref	NA		Ref	NA		Ref	NA
40–59	66 (7)	43 (65)	1.03	0.54–1.95		0.86	0.30–2.42		1.07	0.55–2.08
≥60	64 (7)	53 (83)	2.71	1.27–5.78		2.23	0.73–6.88		2.86	1.32–6.19
Health care settings										
Age, y										
<3	25 (2)	13 (52)	0.46	0.16–1.29		0.50	0.13–1.98		0.75	0.25–2.22
3–19	12 (1)	6 (50)	0.41	0.11–1.52		0.23	0.04–1.19		0.58	0.14–2.41
20–39	44 (4)	31 (71)	1 (Ref)	NA		1 (Ref)	NA		1 (Ref)	NA
40–59	119 (11)	107 (90)	3.73	1.54–9.05		3.42	1.20–9.72		4.59	1.75–12.09
≥60	870 (81)	828 (95)	8.39	4.07–17.29		8.37	3.55–19.78		9.62	4.38–21.12
Sex										
M	965 (49)	716 (74)	1.23	0.96–1.56		1.26	0.92–1.74		1.22	0.95–1.58
F	987 (51)	750 (76)	Ref	NA		Ref	NA		Ref	NA
			Estimate	SE		Estimate	SE		Estimate	SE
Variation between clusters§	NA	NA	0.053	0.089		0.062	0.101		NA	NA
Intraclass correlation coefficient	NA	NA	2.6%	NA		1.95%	NA		NA	NA
Dispersion parameter	NA	NA	0.99	NA		0.99	NA		1.01	NA

*Sensitivity analysis 1 was a multilevel logistic regression model that included only wards and clinics with ≥5 patients. Sensitivity analysis 2 was a logistic regression model (without random effect) that included only the first patient per month within each ward and clinic. GII, genogroup II; OR, odds ratio; Ref, referent; NA, not applicable. An OR>1 indicates an increased risk for norovirus GII.4 infection compared with patients 20–39 y of age. †Proportions for community settings do not add up to 100% because of rounding. ‡Proportion of patients infected with norovirus GII.P4 or GII.4 in each age or sex group. §Estimated to be 1.12 (SE 0.18) in the random intercept-only model (i.e., without the effect of age, sex, and setting).

## Discussion

In this study of 3,846 patients who were positive for NoV by routine diagnostic procedures for gastroenteritis in Denmark during 2006–2010, we detected an association between an age ≥60 years and infection with NoV GII.4 in patients from community and health care settings. We also found that NoV GII.P21/GII.3 was more prevalent in children than in adults, and NoV GI was more frequently detected in patients from foodborne outbreaks than in patients from community and health care settings.

NoV GII.4 was the most prevalent genotype among patients in health care settings (in 91%). However, only 54% of patients from community settings were infected with this genotype. This finding is consistent with findings in a recent study from the United States, which reported that 84%–87% of outbreaks in hospitals and long-term care facilities were caused by GII.4 compared with 17%–75% in other settings (*13*). The reason for the predominance of GII.4 in health care settings has been debated. Patient characteristics, such as increased susceptibility caused by concurrent illnesses or older age, have been suggested (*3*). Virus characteristics, such as greater inherent virulence or increased virus shedding, thereby facilitating transmission in settings with a high concentration of persons, have also been suggested as contributing factors (*2**,**23*). A study by Vega et al. reported that older age was associated with GII.4 outbreaks in diverse settings, such as schools, restaurants, and hospitals (*13*). Our study confirmed this association, which was present in community and health care settings and could partly explain the predominance of GII.4 in hospital settings. The association was stronger for patients in health care settings than for patients in community settings, but the reason for this difference is unknown. Once introduced into a hospital setting, NoV GII.4 might be more easily transmitted than other genotypes, thus infecting elderly patients already hospitalized for other reasons (*2*).

Other studies compared the clinical manifestations of infection with NoV GII.4 with those of infection with other NoV genotypes. Two studies of persons in nursing homes showed that symptoms were more severe in persons infected with GII.4 than in persons infected with other NoV genotypes (*24**,**25*). This finding could be caused by the age of the participants, a finding consistent with the results of our study. A study of NoV infections in newborns, whom the authors presumed to have no pre-existing immunity, showed that the length of symptomatic NoV infection was longer when newborns were infected with NoV GII.4 than with other genotypes (*23*). This finding differs from the hypothesis that only elderly persons have more severe infections when infected with NoV GII.4 than with other genotypes. Because an association between persons ≥60 years of age and infection with NoV GII.4 was also observed in patients from community settings in our study, the increased duration of symptomatic NoV GII.4 infection in infants could be caused by immaturity of their immune systems. Desai et al. conducted a literature review of published NoV outbreaks and concluded that in community settings and long-term care facilities, incidence of hospitalization and death was increased during infection with NoV GII.4 compared with non–GII.4 NoV (*26*). These findings support the theory that the high proportion of GII.4 in hospital settings is caused by viral characteristics rather than host characteristics. However, Desai et al. did not control for age, which might have biased the results toward an increased risk for hospitalization during infection with NoV GII.4.

We determined that NoV GI was more prevalent in foodborne outbreaks than in outbreaks in health care and community settings, which is consistent with previous studies that reported that GI is more frequently observed in foodborne NoV outbreaks than in outbreaks involving person-to-person transmission (*2**,**3*). Excluding GII.4, the proportion of GI was similar in health care and community settings.

When we grouped GII.3 and GII.P21, we observed a higher proportion of these genotypes in young children than among patients ≥60 years of age. This finding is consistent with those of other studies, which indicate that GII.P21 (formerly classified as GII.b) and GII.3 infect mainly young children (*10**,**27**–**29*). It has been hypothesized that genotypes that preferentially infect young children, such as GII.3, require less antigenic variation because of the constant renewal of the host population with persons who do not have established immune-associated protection from previous infections (*30*). This situation is in contrast to that for GII.4, which infects a large proportion of the adult population and thus requires a constant change in host-binding receptors to evade the immune response.

Our study had several limitations. First, sampling bias was caused by inclusion of samples collected for routine diagnostic virus analyses, rather than a cohort encompassing all cases of acute gastroenteritis. Generally, only a few patients in hospital outbreaks of infectious gastroenteritis in Denmark are diagnosed by laboratory testing. We assume that most hospitalized patients with community-acquired NoV infection are tested for NoV as a differential diagnosis to bacterial causes of gastroenteritis, but this assumption might not be correct. As in another study (*31*), our study had an overrepresentation of young and old persons. This overrepresentation could have affected the outcome because it is likely that severity of disease, concurrent illnesses, and young age increase the probability of seeking medical attention. If elderly persons are more likely to be tested for NoV and GII.4 is more virulent than other NoV genotypes, the statistical results could be biased toward an increased effect of age on the odds of infection with GII.4. The best way of testing this hypothesis would be to conduct a cohort study that included all patients with gastroenteritis.

Second, genotyping of NoV isolates was only performed for selected number of NoV-positive patients, but these samples represented almost 90% of all wards, GPs, and outpatient clinics submitting samples to the laboratory. The distribution of age differed between patients whose sample isolates had an assigned genotype. However, a notable finding was the association between NoV GII.4 and age ≥60 years old in community settings. The proportions of genotyped samples did not differ between patients ≥60 and <3 years of age in community settings. Furthermore, we detected 22 NoV capsid and 15 polymerase genotypes, which made it unlikely that problems with laboratory methods systematically biased the results.

Third, we did not have epidemiologic data to compare the distribution of NoV genotypes at an outbreak level. Alternatively, the association between age and NoV GII.4 was examined by including the random effect of clinics and wards. This feature was possible because of the large number of available clinics and wards (*32*). The association between NoV GII.4 and age was also observed in sensitivity analyses that included 1 patient per clinic or hospital ward in each calendar month or included clinics and wards with ≥5 patients.

Fourth, we estimated that most hospitalized patients were nosocomially infected with NoV. This estimate was based on admission and sampling dates because clinical data were not available. For some patients, sampling may have been performed >1 day after onset of symptoms. Therefore, the proportion of nosocomially infected patients might have been overestimated.

In this retrospective study of NoV gastroenteritis in Denmark, we compared infections in patients from foodborne outbreaks, community settings, and health care settings. Our results confirmed that most NoV genotypes circulating in health care settings were GII.4 and that infection with NoV GII.P21 or II.3 was more prevalent in children than adults. We observed an association between older age and infection with NoV GII.4, which could partly explain why most NoV infections in health care settings are caused by this genotype. Cohort studies testing this hypothesis would be of value.

Technical Appendix PCR conditions used in determination of norovirus epidemiology in community and health care settings and relationship to patient age, Denmark, 2006–2010.
