# Supplementary material for: Norovirus Epidemiology in Community and Health Care Settings and Association with Patient Age, Denmark
Source: Emerg Infect Dis. 2014 Jul;20(7):1123–31. doi: 10.3201/eid2007.130781 (PMC4073851; doi:10.3201/eid2007.130781)
Supplement: Technical Appendix — PCR conditions used in determination of norovirus epidemiology in community and health care settings and relationship to patient age, Denmark, 2006–2010. [file 13-0781-Techapp-s1.pdf]

Article DOI: http://dx.doi.org/10.3201/eid2007.130781

Norovirus Epidemiology in Community and Health Care Settings and Association with Patient Age, Denmark

Technical Appendix

PCR Conditions Used in Determination of Norovirus Epidemiology in Community and Health Care Settings and Association with Patient Age, Denmark

Real-Time Quantitative Reverse Transcription PCR (RT-PCR) Cycling Conditions

PCR conditions for the MX3000-MX3005 system (Stratagene, La Jolla, CA, USA) were incubation at 50°C for 20 min, activation at 95°C for 15 min, and 40 amplification cycles of denaturation at 95°C for 15 s and annealing/extension at 50°C for 1 min.

Polymerase Gene RT-PCR Cycling Conditions

Primers JV12Y-JV13H and JV12BH-NVp110 were used. PCR conditions were incubation at 50°C for 30 min, activation at 95°C for 15 min; 40 amplification cycles of denaturation at 94°C for 30 s, annealing at 37°C for 30 s, and extension at 72°C for 30 s; and a final elongation at 72°C for 10 min.

In some instances, nested PCR was performed. First-round nested RT-PCR was performed with primers NV32, NV32a, and NV36. PCR conditions were incubation at 42°C for 30 min, activation at 94°C for 15 min; 35 amplification cycles of denaturation at 94°C for 30 s, annealing at 42°C for 30 s, and extension at 72°C for 45 s; and final elongation at 72°C for 10 min. Second-round nested RT-PCR was performed with primers NV33, NV33a, NV35, and NV35a. PCR conditions were activation at 95°C for 10 min; 35 amplification cycles of denaturation at 95°C for 30 s, annealing at 48°C for 30 s, and extension at 72°C for 2 min; and final elongation at 72°C for 5 min. PCR products were examined for correct size by electrophoresis in agarose gels containing 1% ethidium bromide.

Capsid Gene RT-PCR

These conditions were used with genotype I (GI) and GII primer sets. First-round PCR conditions were incubation at 42°C for 60 min, activation at 95°C for 15 min, 45 amplification cycles of denaturation at 95°C for 60 s, annealing at 41°C for 60 s, and extension at 72°C for 60 s; and a final elongation at 72°C for 10 min. Second-round nested RT-PCR conditions were activation at 95°C for 10 min; 40 amplification cycles of denaturation at 95°C for 30 s, annealing at 48°C for 30 s, and extension at 72°C for 2 min; and a final elongation at 72°C for 5 min. PCR products were examined for correct size by electrophoresis in agarose gels containing 1% ethidium bromide.
